# Editorial Department

**Published:** 1857-12

**Authors:** Reuben Littick


The following communication we publish verbatim et literatim, hoping that it may find an owner, or if not, that some of our readers will give the writer the benefit of his judgment in the treatment of his case, which is utterly beyond our comprehension. The direction is sufficiently plain, but our worthy senior editor disclaims all connection or relation with it. If there be any other Dr. Hunt who has claims to this epistle, he can have the original by "proving property and paying charges." F.

The letter is plainly directed to

Mr. Dr. Hunt, Buffellow, Buffellow, Newyork.

Delaware October the 15 1857

Dear Sir

I take this oppetunity to wite to you that we have given your meicin a triel and it proves to no Efect, the girle took all her medicen and it maid her faces woise and the medicen you left for my wife she took one or two pouders and was obtige to stop takaig them; and the surip the Drugest said that the Chips you Recomended to be put in the sirep that it would not answer to take for meicen tharefore we Did not make Eny.

they took sum of your sasafrella and it Didnot Do Eny good; and tharefour I Canot pay For nothing and more than that I am not able fore the Reason that the hale storme Disstroyed most all my Crop

Your Respectfly &c

REUBEN LITTICK.

